# Older adults lack SARS CoV-2 cross-reactive T lymphocytes directed to human coronaviruses OC43 and NL63

**DOI:** 10.1038/s41598-020-78506-9

**Published:** 2020-12-08

**Authors:** Giulietta Saletti, Thomas Gerlach, Janina M. Jansen, Antonia Molle, Husni Elbahesh, Martin Ludlow, Wentao Li, Berend-Jan Bosch, Albert D. M. E. Osterhaus, Guus F. Rimmelzwaan

**Affiliations:** 1 Research Center for Emerging Infections and Zoonoses, University of Veterinary Medicine, Bünteweg 17, 30559 Hannover, Germany; 2 Virology Section, Infectious Diseases and Immunology Division, Department of Biomolecular Health Sciences, Faculty of Veterinary Medicine, Utrecht University, Utrecht, The Netherlands

**Keywords:** SARS-CoV-2, Viral host response

## Abstract

Currently, infections with SARS-Coronavirus-2 (SARS-CoV-2), the causative agent of the COVID-19 pandemic, are responsible for substantial morbidity and mortality worldwide. Older adults subjects > 60 years of age account for > 95% of the over one million fatal cases reported to date. It is unclear why in this age group SARS-CoV-2 infection causes more severe disease than in young adults. We hypothesized that differences in SARS-CoV-2 cross-reactive cellular immunity induced after infection with human coronaviruses (HCoVs), like OC43 and NL63, were at the basis of the differential mortality (and morbidity) observed after SARS-CoV-2 infection, because a small proportion of HCoV-specific T cells cross-react with SARS-CoV-2. Our data demonstrate that pre-existing T cell immunity induced by circulating human alpha- and beta-HCoVs is present in young adult individuals, but virtually absent in older adult subjects. Consequently, the frequency of cross-reactive T cells directed to the novel pandemic SARS-CoV-2 was minimal in most older adults. To the best of our knowledge, this is the first time that the presence of cross-reactive T cells to SARS-CoV-2 is compared in young and older adults. Our findings provide at least a partial explanation for the more severe clinical outcome of SARS-CoV-2 infection observed in the elderly. Moreover, this information could help to design efficacious vaccines for this age group, aiming at the induction of cell-mediated immunity.

## Introduction

Severe Acute Respiratory Syndrome coronavirus 2 (SARS-CoV-2) is the causative agent of COVID-19. The first cases of SARS-CoV-2 infection were reported in December 2019 and ever since the virus spread rapidly around the globe, causing mild to severe respiratory disease, which prompted the World Health Organization to declare the COVID-19 outbreak a pandemic on March 11th. As of October 8, more than 35,000,000 cases have been reported, of which more than 1,000,000 had a fatal outcome (https://www.who.int/emergencies/diseases/novel-coronavirus-2019).

People older than 60 years of age are especially at a high risk for developing severe disease with fatal outcome (https://www.cdc.gov/nchs/nvss/vsrr/covid_weekly/index.htm#AgeAndSex). In fact, over 95% of all deaths caused by SARS-CoV-2 infection were reported to be in this age group^1,2^.

The reason why SARS-CoV-2 infection causes more severe disease in older adults is largely unknown, but most likely has an immunological basis.

On the one hand, it is known that older adults do not respond to infection or vaccination as well as younger individuals, a phenomenon known as immunosenescence that entails defects in the innate and adaptive immune responses^3,4^.

On the other hand, variation in immune imprinting might account for the observed differences in susceptibility to SARS-CoV-2 infections. It has been shown that infection with human coronaviruses (HCoVs), like OC43, HKU-1 (beta coronaviruses), NL63 and 229E (alpha coronaviruses) can induce virus-specific CD4^+^ and CD8^+^ T cell responses that display cross-reactivity with the novel emerging coronavirus SARS-CoV-2, although to a limited extent^5,6^. In some studies however, SARS-CoV-2 specific T cells could not be demonstrated in non-exposed individuals^7^. Thus, the existence of SARS-CoV-2 cross-reactive T cells directed to HCoVs is still a matter of debate. Of interest, in patients with non-lethal COVID-19, potent virus-specific CD4^+^ and especially CD8^+^ T cell responses were observed prior to recovery from disease, indicating that cell-mediated immunity to SARS-CoV-2 is a correlate of protection^5–8^.

A role of cell-mediated immunity in the control of 2003 SARS-CoV infection and protective immunity was demonstrated in mouse models^9,10^.

We hypothesized that the level of pre-existing cross-reactive T cell immunity correlates with the differences in disease outcome observed between the age groups. A higher level could be the cause of T cell hyper-responsiveness in the lungs of infected individuals and thus contributing to pathogenesis of COVID-19. This has been observed for infection with influenza viruses predominantly in mouse models, where production of TNF-α by virus-specific T cells may have detrimental effects, either directly or through the induction of chemokines such as MIP-2 or MCP-1 by alveolar cells, resulting in the recruitment of pro-inflammatory cells^11^.

In contrast, the presence of pre-existing cross-reactive T cells could contribute to protective immunity as observed for other respiratory infections, in particular influenza viruses^12–14^. Reduced levels of cross-reactive memory T cells in older adults could result in inadequate control of SARS-CoV-2 infection and may explain the differential age distribution of fatal cases.

To test this hypothesis, we used peripheral blood mononuclear cells (PBMCs) obtained from healthy blood donors before the COVID-19 outbreak, that were divided into two age groups: young adult (19–27 years of age) and older adults (61–70 years of age). The T cell immunity to HCoVs OC43 and NL63 and the cross-reactivity with SARS-CoV-2 was then assessed and compared between these two groups.

First, we demonstrated that subjects of the younger age group displayed strong T cell responses to the HCoVs and that a proportion of these T lymphocytes indeed displayed cross-reactivity with SARS-CoV-2.

Second, we demonstrated that T cell memory lymphocytes to HCoVs OC43 and NL63 were virtually absent in the group of older adults and that accordingly SARS-CoV-2 cross-reactive T cells were absent. This may explain, at least in part, the poor disease outcome in older adults and suggests that prophylactic vaccines should induce virus-specific cell mediated immunity in addition to virus neutralizing antibodies.

## Results

### HCoV-specific T cells and antibodies in young and older adults

To assess the frequency of T cells specific for HCoVs causing predominantly seasonal “common cold”, we utilized the IFN-γ ELISpot assay. PBMCs obtained from forty-four healthy blood donors (Table 1 and Supplementary Table S1) divided into two age groups, young adults (N = 23) and older adults (N = 21) were stimulated with inactivated HCoV-NL63 and HCoV-OC43 or left unstimulated.

**Table 1:** Demographic characteristics of the study cohort.

	Young adults	Older adults
Number of subjects	23	21
Age (yr; median ± SD)	22	64
Age (yr; range)	19–27	61–70
**Sex, n. (%)**		
Male	12 (51.2)	18 (85.7)
Female	11 (47.8)	3 (13.7)

**Figure 1:**
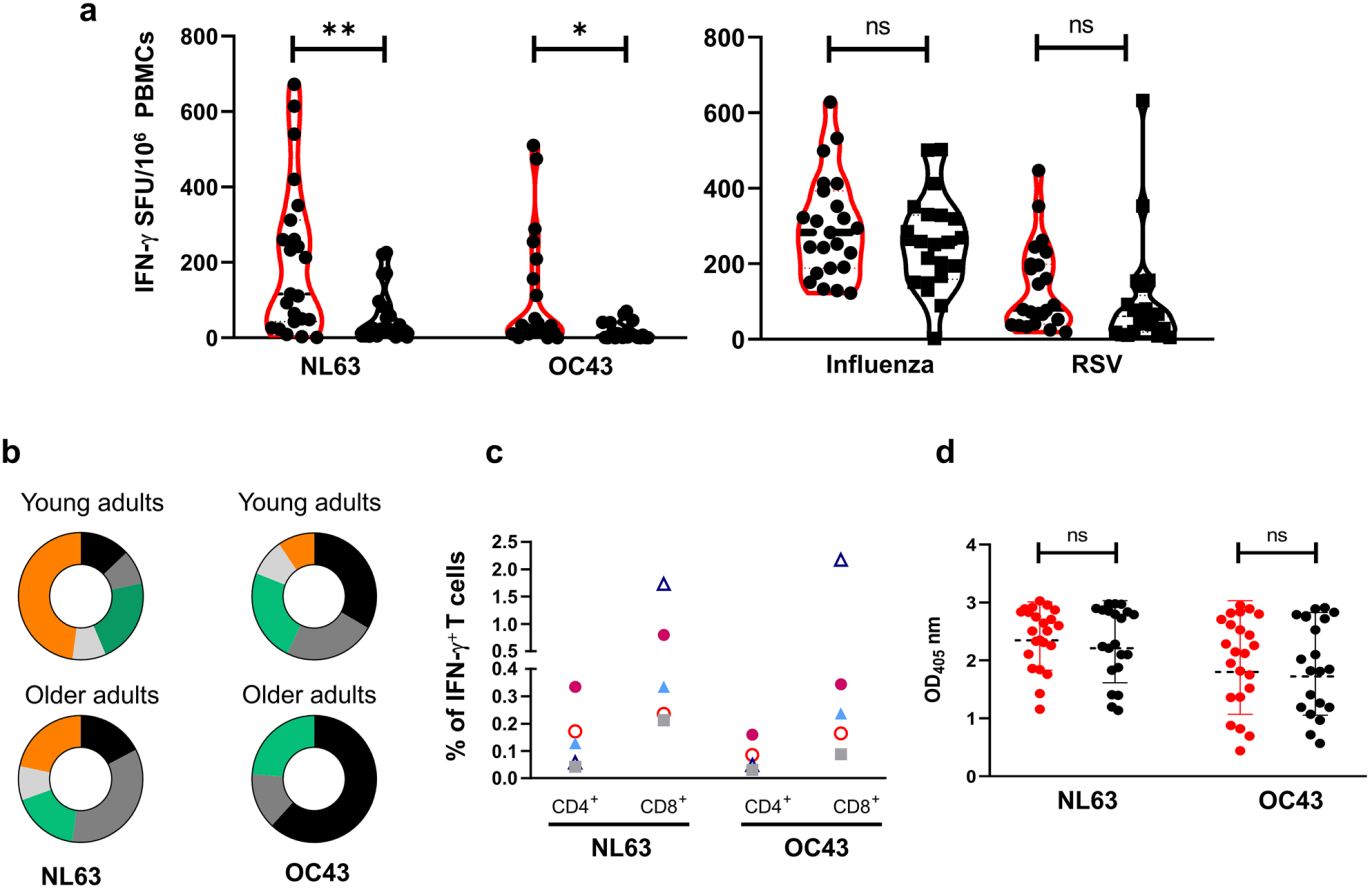
HCoV-NL63 and HCoV-OC43-specific immunity in young and older adults. (**a**) HCoV-specific T cells response measured as frequency of IFN-γ spot forming units (SFU) after stimulation of PBMCs isolated from young (n = 23; red outline) and older adults (n = 21; black outline) with inactivated HCoV-NL63 and HCoV-OC43. As controls, PBMCs were stimulated with live RSV or inactivated influenza virus vaccine (VaxigripTetra, Sanofi Pasteur). Data were background-subtracted and expressed as SFU/10^6^ PBMCs. Dotted lines represented the median. (**b**) Magnitude of the IFN-γ response among young and older adults expressed as percentage of individuals showing < 10 (black), 10–30 (dark grey), 30–100 (green), 100–200 (light grey) and > 200 (orange) SFU/10^6^ PBMCs. (**c**) Ex vivo IFN-γ producing CD4^+^ and CD8^+^ T cells in five selected high-responders upon ex-vivo stimulation of PBMCs with inactivated NL63 and OC43 measured by ICS. The data are expressed as percentage of IFN-γ^+^ CD4^+^ or CD8^+^ T cells. (**d**) Serum NL63- and OC43-specific IgG antibodies in young (red dot) and older (black dot) adults measured by ELISA. Each dot and symbol represent a single subject. Statistical comparison between the two groups was performed using non-parametric Mann–Whitney test for unpaired samples (**p < 0.005; *p < 0.05; ns: not significant).

The frequency of HCoV-specific T cells secreting IFN-γ in young adults was significantly higher than that in older adults for both NL63 (116 ± 201 vs 24 ± 74; *p* < 0.005) and OC43 (24 ± 149 vs 5 ± 22; *p* < 0.05) (Fig. 1a) and overall, stimulation with NL63 induced a stronger response compared to OC43. Because there was an unequal distribution between males and females in the two age groups, we re-analyzed the data only using the male subjects and confirmed that older subjects displayed a lower response than young adults (NL63 *p* < 0.005; OC43 *p* < 0.05) (Supplementary Fig. S1). The proportion of study subjects giving a positive response (> 10 SFU per million PBMCs) was higher in young adults than in the older adults (87% young vs 66.7% older for NL63; 83% vs 39% for OC43) (Fig. 1b). Moreover, the majority of the young subjects showed a frequency of NL63-specific response above 100 SFU per million PBMCs (56.5% young vs 19% older adults), with 47.8% of them having more than 200 SFU/million PBMCs (Fig. 1b). The same trend was observed for the OC43-specific response. None of the older subjects had a frequency greater than 100 SFU/million PBMCs compared to the 30.4% found in the younger subjects. Of note, these differences were not observed after stimulation with Respiratory Syncytial virus (RSV), influenza virus or non-specific stimulation with anti-CD3 antibody (Fig. 1a and Supplementary Table S2).

We then sought to identify the T cell subsets responsible for IFN-γ secretion in response to the inactivated HCoV-NL63 and HCoV-OC43 preparations by flow cytometry. The very low frequency of HCoVs-specific T cells, precluded detection of virus-specific T cells by intracellular IFN-γ staining. Therefore, we selected five high-responders (HR) from the young age group and restimulated their PBMCs ex-vivo with the inactivated HCoV preparations. Although the magnitude of the response varied, all young HR subjects tested had virus-specific CD4^+^ and CD8^+^ IFN-γ^+^ T cells. Thus, the inactivated HCoVs preparations induced IFN-γ production in CD4^+^ and CD8^+^ T cells (Fig. 1c).

To determine whether the lower frequency of virus-specific T cells found in the older adult study subjects was caused by differences in exposure rates to HCoVs, we examined the NL63 and OC43-specific IgG antibodies by ELISA. We found that young and older adults had similar levels of NL63- and OC43-specific antibodies, with a lower level to OC43 (Fig. 1d), suggesting that other factors accounted for the lower frequency of HCoVs-specific T cells in older adult subjects.

Taken together, these data indicated that a significant number of older adults did not show an in vitro recall T cell response to the HCoVs tested, and that the magnitude of such response was lower when compared to that of young adult subjects.

### Frequency of S protein-specific T cells in young and older individuals

**Figure 2:**
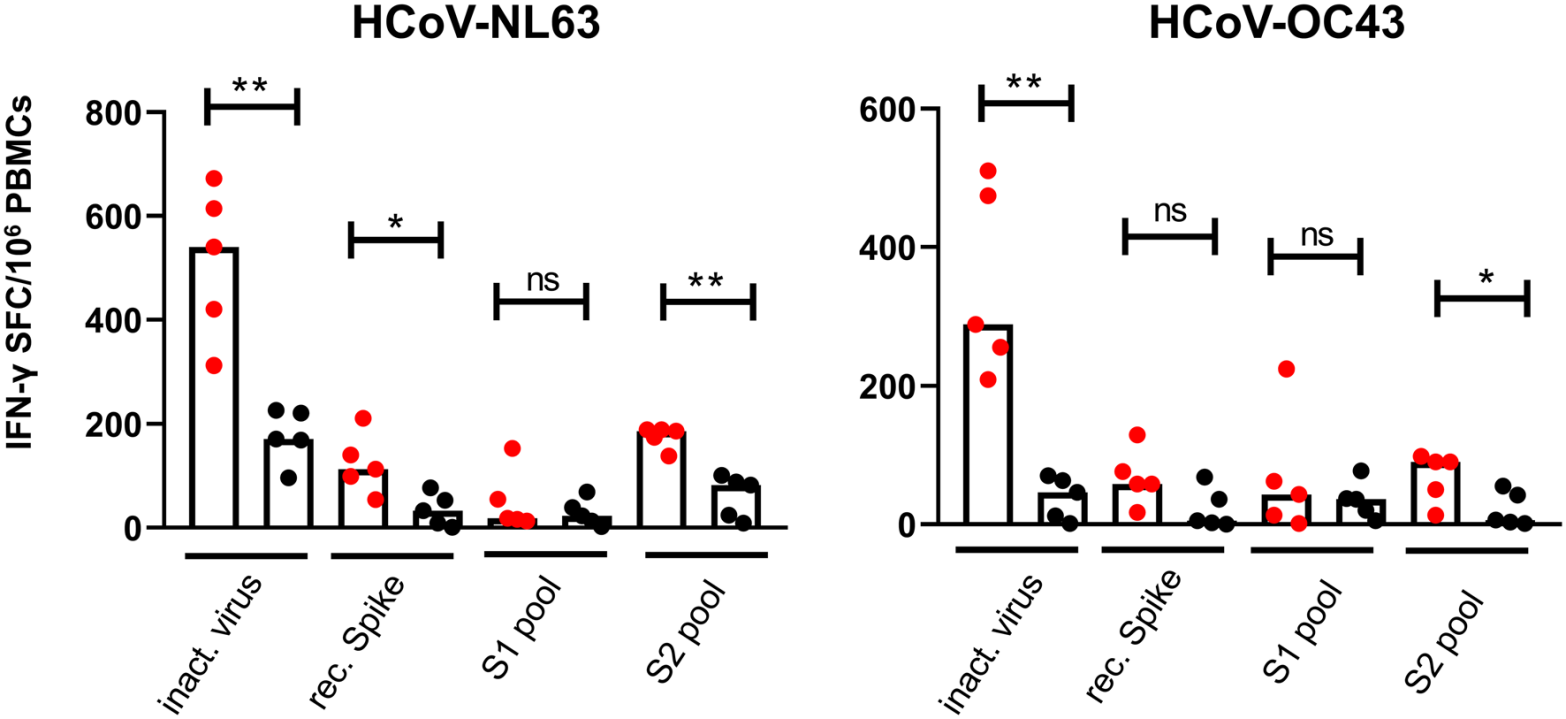
S protein-specific T cell immune responses in young and older subjects. PBMCs from HCoVs high-responders, 5 young (red dot) and 5 older (black dot) adults, were cultured in the presence of NL63 and OC43 inactivated virus (inact. virus; 2.5 µg/ml) recombinant spike proteins (rec. Spike; 2.5 µg/ml) or peptide pools (S1 and S2; 0.4 µg/ml each peptide) and frequency of IFN-γ producing T cells measured by ELISpot. Data were background-subtracted and expressed as SFC/10^6^ PBMCs. Each dot represents a single subject. Statistical comparison between the two groups was performed using non-parametric Mann–Whitney test for unpaired samples (**p = 0.0079; *p < 0.05; ns: not significant).

To confirm our data obtained with the inactivated NL63 and OC43 HCoV preparations, and further characterize the differential response observed in young and older individuals, we sought to measure the T cell response to the HCoVs Spike (S) surface glycoprotein. PBMCs from ten subjects (5 young and 5 older adults) with high IFN-γ response to inactivated HCoVs (HR), were stimulated with NL63 and OC43 rS proteins or peptides spanning the entire S protein and divided in two pools (S1 and S2) (Supplementary Tables S3 and S4). In line with the data obtained with the inactivated HCoV preparations, NL63 rS protein and peptides both induced higher frequency of IFN-γ producing T cells compared to those derived from OC43 (Fig. 2). However, the rS protein-specific response was of lower magnitude than the one obtained with the inactivated HCoVs (NL63: median 540 vs 113 young adults and 171 vs 33 older adults; OC43: 288 vs 58 young and 46 vs 5 older adults). This latter finding suggests that inactivated HCoVs activated T cells directed toward other viral proteins in addition to those specific for the S protein. Interestingly, a comparably low-frequency of virus-specific T cells were observed in both age groups with the S1 peptide pool. In contrast, a higher frequency of NL63- and OC43-specific T cells was observed in younger subjects compared to older subjects with the S2 peptide pool (*p* < 0.05).

These data suggest that, older adults displayed a lower frequency of S protein-specific IFN-γ secreting T cells compared to young individuals. This difference was more pronounced when NL63-rS protein and S2 peptide pool were used for ex vivo stimulation.

### HCoVs induced cross-reactive T cells to SARS-CoV-2

**Figure 3:**
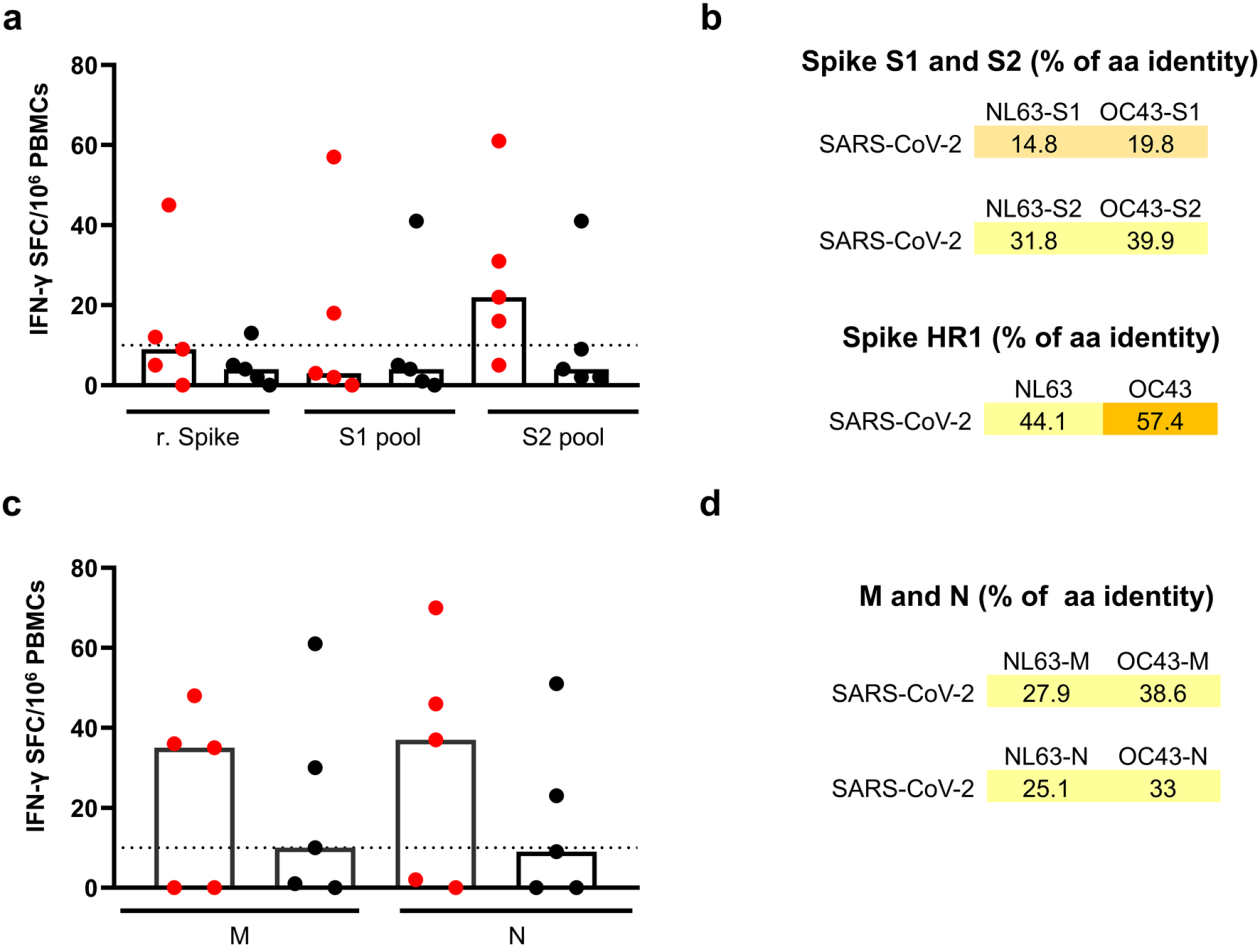
SARS-CoV-2 S, M and N protein cross-reactive T cells in high-responders. PBMCs from 5 young (red dot) and 5 older (black dot) adults high-responders, were cultured in the presence of (**a**) SARS-CoV-2 recombinant Spike protein (2.5 µg/ml) or S1 and S2 peptide pools (1 µg/ml each peptide); (**c**) N and M peptide pools (1 µg/ml each peptide) and frequency of IFN-γ producing T cells measured by ELISpot. Data were background-subtracted and expressed as SFC/10^6^ PBMCs. Each dot represents a single subject and columns the median. Percentage of amino acids homology between the SARS-CoV-2 and HCoV NL63 and OC43 for: (**b**) S1, S2 and heptad repeat 1 (HR1), (**d**) M and N.

The presence of cross-reactive T cells in a small portion of SARS-CoV-2 unexposed individuals with pre-existing immunity to HCoVs has recently been documented^5^. We have shown that older adult subjects displayed a lower frequency of HCoV-NL63 and OC43 specific T cells as compared to young adult study subjects. Therefore, we investigated the presence of SARS-CoV-2 cross-reactive T cells in young and older subjects using the IFN-γ ELISpot assay. Due to its sensitivity, the ELISpot can detect very low frequencies of antigen-specific T cells that are difficult to detect with other methods, including flow cytometry. The PBMCs of ten high-responders (5 young and 5 older adults), the same used to characterize the T cell immunity to HCoV, and nine non-responders (NR) (4 young and 5 older adults) were stimulated with rS protein and peptide pools spanning the entire sequence of SARS-CoV-2 S, M and N proteins. The S protein peptides were divided into two pools spanning the N-terminal (S1; 1–673 aa residues) and C-terminal (S2; 633–1273 aa residues) domains. Stimulation of PBMCs with the SARS-CoV-2 rS protein gave a positive response in one out of the five older adults and two out of the five young adult subjects tested (Fig. 3a). Similar results were obtained using the S1 peptide pool, although the magnitude of the response was higher than observed with the rS protein. Interestingly, stimulation with the S2 peptide pool, showed a positive response in four and one out of the five young adult and older adult subjects, respectively (median 22 vs 9 SFC/10^6^ PBMCs). Compared to the S1 region, the S2 region of SARS CoV-2 S protein displays a higher degree of sequence homology with HCoVs, which could explain the higher degree of T cell cross-reactivity (Fig. 3a,b). In this respect, we have identified a region in the S2 domain, corresponding to the heptad repeat 1 (HR1), with more than 50% homology between SARS-CoV-2 and HCoVs NL63 and OC43 (Fig. 3b).

We also examined the reactivity with SARS-CoV-2 M and N proteins, using peptide pools (Fig. 3c). Using PBMCs of HR young and older adult subjects, we detected SARS-CoV-2 M- (35 and 10 SFC/10^6^ PBMCs in young and older adults respectively) and N- (37 and 9 SFC/10^6^ PBMCs in young and older adults, respectively) protein-specific T cells (Fig. 3c). The HCoV OC43 exhibited a higher amino acid sequence homology with SARS-CoV-2 M and N (38.6% and 33% for M and N, respectively) than NL63 HCoV (27.9% and 25.1% for M and N, respectively) (Fig. 3d).

**Figure 4:**
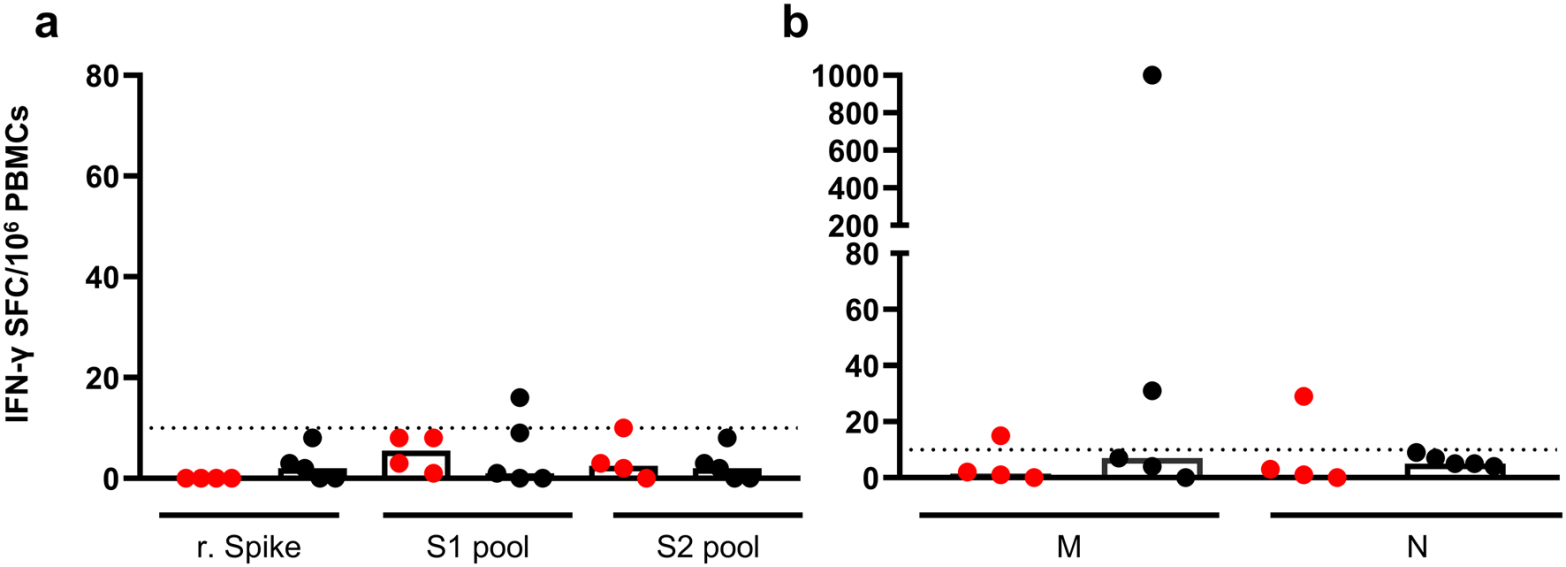
SARS-CoV-2 S, M and N protein cross-reactive T cells in non-responders. PBMCs from 4 young (red dot) and 5 older (black dot) adults NR, were cultured in the presence of (**a**) SARS-CoV-2 recombinant Spike protein (2.5 µg/ml) or S1 and S2 peptide pools (1 µg/ml); (**b**) N and M peptide pools (1 µg/ml each peptide) and frequency of IFN-γ producing T cells measured by ELISpot. Data were background subtracted and expressed as SFC/10^6^ PBMCs. Each dot represents a single subject and columns the median.

PBMCs from nine non-responding (NR) study subjects (4 young and 5 older adults) that tested negative in the IFN-γ ELISpot assay upon stimulation with HCoVs NL63 and OC43, did not respond to the SARS-CoV-2 rS protein. One subject out of the nine showed a low response to the S1 peptide pool, with a frequency slightly above the cut-off (Fig. 4a). The response to the M and N peptide pools in these selected NR study subjects was not detectable or very low (Fig. 4b). One older adult NR subject however displayed a high response to the SARS-CoV-2 M peptide pool (> 1000 SFU/10^6^ PBMCs).

Our data confirm the presence of cross-reactive T cells to SARS-CoV-2 in study subjects unexposed to the newly emerging coronavirus but with pre-existing immunity to the HCoVs NL63 and OC43. Of special interest, the frequency of HCoV- specific T cells in older adults was significantly lower than in young adults suggesting that also the frequency of SARS-CoV-2 cross-reactive T cells is lower in this age group.

## Discussion

Our study demonstrates that the frequency of T cells specific for HCoVs is lower in older adults compared to young adults and consequently, pre-existing cross-reactive cell mediated immunity to the newly emerged SARS-CoV-2 is impaired in this age group. The lack of this cross-reactive cell mediated immunity may partially explain the severe course of disease upon infection with SARS-CoV-2 in older adults, which often has a fatal outcome in this age group.

In March 2020 the WHO declared the global outbreak of respiratory disease caused by the zoonotic human coronavirus SARS-CoV-2 a pandemic (https://www.euro.who.int/en/health-topics/health-emergencies/coronavirus-covid-19/news/news/2020/3/who-announces-covid-19-outbreak-a-pandemic). Ever since, the SARS-CoV-2 has caused, and continues to cause, vast numbers of infections worldwide, which can have a fatal outcome especially in patients that are over 60 years old. It has been shown that reduced numbers of CD4^+^ and CD8^+^ T cells in patients admitted to the intensive care correlated with COVID-19-associated severity and mortality, suggesting that T cells are important for a favorable clinical outcome^15,16^. Data supporting the role of T cells are also coming from studies carried out during the 2003 outbreak of infections with SARS-CoV (hereafter SARS-CoV-1), which is a beta-coronavirus closely related to SARS-CoV-2^17^. Data on duration of the T cell immunity after infection with SARS-CoV-2 are not yet available, but in SARS-CoV-1 recovered patients, virus-specific T cells were detectable in the blood more than a decade after infection suggesting long-lasting immunity conferred by T cells, while virus-specific antibodies waned rapidly^18–21^.

Recent studies have demonstrated pre-existing cross-reactive T cells to SARS-CoV-2 in unexposed individuals, presumably induced after infection with one or more of the HCoVs^5,6,21,22^. Furthermore, memory CD8^+^ T cells cross-reactive with SARS-CoV-1 have been demonstrated in a small portion of non-exposed healthy individuals, after in vitro recall of virus-specific T cells with an inactivated SARS-CoV-1 virus preparation^23^. In contrast, serum antibodies from endemic HCoVs pre-exposed subjects did not cross-react with SARS-CoV-2^24^.

In the present study, for the first time, HCoV-specific T cell immunity was investigated in study subjects of different age groups, young and older adults. The frequency of NL63- and OC43-specific T cells was significantly lower in the group of older adults. Some of these cells cross-reacted with the SARS-CoV-2 S, M and N proteins, although the study subjects never had been exposed to this newly emerged coronavirus. Of note, the proportion of males in the older adult group was higher than in the group of young adults. This is relevant regarding the higher susceptibility of older males to infection with SARS-CoV-2. However, we have re-analyzed the ELISpot data obtained with NL63 and OC43, only using the male subjects from both age groups and confirmed that older adults displayed a lower response than young adults, independent of the gender (NL63 *p* < 0.005; OC43 *p* < 0.05).

The majority of the S protein-specific cross-reactive T cells were directed to the S2 region (C-terminus) of the protein, which displays a relatively high amino acid sequence homology between SARS-CoV-2 and the HCoVs (31.8% for NL63 and 39.9% for OC43). Deconvolution of the S2 peptides pool and identification of cross-reactive epitopes in S2, would be useful for rational design of vaccines that aim at inducing cross-reactive T cell responses. S protein-specific T cell responses were also found to be dominant after infection with SARS-CoV-1^25–27^. Moreover, we also demonstrated that M and N proteins represent a target for cross-reactive T cells.

Importantly, in study subjects in which HCoV-specific T cell were absent, including the majority of the older adults (Non-responders, < 10 SFC/10^6^ PBMC), no or very low numbers of SARS-CoV-2 specific T cells were demonstrated with one exception.

One subject in the older adults group, who did not respond to stimulation with the HCoVs NL63 and OC43, had a high frequency of T cells to the SARS CoV-2 M protein, (1000 SFC/10^6^ PBMCs). This finding is hard to explain, but the HLA haplotype of this subject (A*02; B*15; B*18) has been associated with highest capacity to present conserved peptides shared among HCoVs^28^. Possibly, M-specific cross-reactive T cells were induced by infection with other “common cold” or unknown coronaviruses not tested in the present study.

It is important to emphasize that cross-reactive CD4^+^ T helper cells specific for structural proteins, could also play an important role in supporting antibody production. The S protein is the target for virus-neutralizing antibodies and the most important component of candidate vaccines currently under development^29^. Moreover, several neutralizing S protein-specific monoclonal antibodies have been described, which are considered for therapeutic or prophylactic treatment^30–32^.

The relatively poor cell mediated immunity to HCoVs and the consequent poor cross-reactivity with SARS-CoV-2 in the vast majority of older adults, may have bearing for their capacity to cope with infection with SARS-CoV-2 and may partially explain the differential age-dependent outcome of infection.

For other respiratory viruses, like influenza A viruses it has been shown that the frequency of pre-existing virus-specific CD8^+^ T cells inversely correlated with the severity of disease after infection with a heterosubtypic influenza virus strain as was demonstrated during the pandemic of 2009 and in numerous animal models^12,33^. Also against various family members of the Flaviridae, cross-reactive T cells have been demonstrated in mice^34–36^. In a mouse model, it was shown that Dengue virus (DENV) specific CD8 + T cells afforded protection against subsequent Zikavirus infection^37,38^. Also human T cells directed to DENV or other flaviruses display a high degree of cross-reactivity^39–41^. However, as for the SARS-CoV-2 cross-reactive T cells, the contribution of flavivirus cross-reactive CD4^+^ and CD8 ^+^ T cells in protecting humans from infection with heterologous flaviviruses remains to be resolved.

It is tempting to speculate that the high frequency of HCoV-specific T cells, of which a proportion is cross-reactive with SARS CoV-2, afford a level of protection in young adults, that is absent in older adults, although many other factors associated with ageing may account for immuno-senescence.

Human coronaviruses are respiratory pathogens accounting for about 10–30% of common cold cases and are endemically transmitted^42,43^. Adults have been repeatedly infected with HCoVs , even with the same strain as was demonstrated in a human challenge study and recently supported by a longitudinal study monitoring reinfections for 35 years^44,45^. It is unclear whether the poor T cell responsiveness in the older adults is the result of repeated infections with HCoVs and exhaustion of virus-specific T cells or a general state of immunosenescence, which has been associated with ageing^46,47^. However, when we tested the T cell immune responses to two other respiratory viruses, RSV and influenza virus, we found no statistically significant differences in the IFN-γ responses between the two age groups, suggesting that impaired immunity in older adults is a HCoV-specific phenomenon.

Alternatively, it cannot be excluded that pre-existing SARS-CoV-2 reactive T-cells, could actually lead to hyperactive responses in the lung upon infection and thus contribute to immunopathogenesis of infection^48^. In this respect, it is of interest to note that late treatment of SARS-CoV-2 patients with dexamethasone, an immunosuppressive agent acting on T cells, had a positive effect on the disease outcome^49^.

Therefore, further investigation on the role of pre-existing immunity to endemic HCoVs on SARS-CoV-2 infection seems warranted.

## Materials and methods

### Study subjects

Blood obtained from forty-four healthy blood donors were used in this study. Two groups of subjects were selected based on their age: young adults (19–27 years of age (yoa) N = 23) and older adults (61–70 yoa, N = 21). The demographic characteristics of the study subjects are summarized in Table 1 and Supplementary Table S1.

For this study, blood collected between April and August 2019 at the Blood bank of the Hannover Medical School (Medizinische Hochschule Hannover, MHH) was used. The use of blood for scientific purposes was approved by the local MHH ethical committee (Permit number 3393–2016) and the subjects gave their written informed consent. The work described here has been carried out in accordance with the code of ethics of the world medical association (declaration of Helsinki).

### Blood samples and PBMC isolation

To obtain serum samples, blood was collected by venipuncture into serum separator tubes (Sarstedt). After centrifugation for 15 min at 1800 × *g*, the serum was collected and frozen at − 20 °C until use.

Peripheral blood mononuclear cells (PBMCs) were isolated from peripheral blood by density gradient centrifugation (Lymphoprep; Stem cell) following the manufacturer’s instructions. After isolation, cells were counted and frozen in 90% fetal bovine serum (FBS, Thermo Fisher), 10% dimethyl sulfoxide (DMSO, Sigma Aldrich), and cryopreserved in liquid nitrogen until use. The PBMCs were thawed in complete RPMI 1640 medium, supplemented with penicillin/streptomycin, Glutamax, vitamins, non-essential aminoacids (all at 1% v/v, Thermo Fisher), 10% (v/v) heat-inactivated Fetal Bovine Serum (FBS; Gibco Thermo Fisher) (R10F) and 50 µg/ml of DNAse (Sigma Aldrich).

### Sequence alignment and homology

Protein sequences (N, M and S) were retrieved from NCBI-database whole genome-sequences (SARS-CoV2: MN908947.3; SARS-CoV: NC_004718.3; NL63: NC_005831.2 and OC43: NC_005147.1). Sequences were chosen based on the peptide pool for T-cell stimulation. The protein sequences were aligned using MUSCLE alignment tool and pairwise identity between sequences was calculated using GeneiousPrime software (version 2019.0.4).

### Reagents for detection of virus-specific T cell activation and serology

#### Virus preparations

HCoV-NL63 and HCoV-OC43 (NL63 and OC43, respectively) were used in this study and belong to the alpha- and beta-coronavirus genus, respectively. Briefly, Vero cells were grown to about 80% confluence and infected with the HCoVs in serum-free media for 1 h at 37 °C. The supernatant was harvested on day seven post-infection and subjected to concentration by ultracentrifugation using a 20% sucrose cushion. The concentrated virus was then inactivated using a proprietary inactivation method based on binary ethylenimine (European Veterinary Laboratory (EVL, Netherlands)^50,51^. A clinical isolate of RSV (RSV-A-0594; kindly provided by Thomas Schulz; Institute of Virology Hannover Medical School) was grown in HEp-2 cells in Opti-MEM (Thermo Fisher Scientific) and titrated using a modified immunoplaque assay as described previously^52^. Virus stocks were stabilized in 32% glycerol, 8% FBS and stored at − 150 °C until use.

The VaxigripTetra (Sanofi Pasteur), a commercially quadrivalent split-virion inactivated influenza vaccine (season 2017/2018), was used for in-vitro restimulation of the PBMCs.

### Recombinant Spike proteins

The S ectodomains of SARS-CoV-2 (residues 1–1213, GenBank accession no. QHD43416.1), HCoV-OC43 (residues 15–1263, UniProtKB: Q696P8) and of HCoV-NL63 (residues 16–1291, UniProtKB: Q6Q1S2) were expressed and affinity-purified from mammalian or insect cells with a C-terminal trimerization motif and StrepTag affinity tag, as previously described^30,53,54^.

### Peptides

Peptides of NL63 (GenPept: Q6Q1S2) and OC43 (GenPept: NP_937950) peptide arrays spanning the entire spike protein of the respective viruses (BEI Resources) were dissolved in cell culture grade DMSO (Hybrimax Sigma). For each array two peptide pools were prepared: S1, covering the N-terminal amino acids until the cleavage site (peptides 1 to 126 for NL63 and 1 to 128 for OC43); S2, from the cleavage site until the C-terminus (peptides 123 to 226 for NL63 and 126 to 226 for OC43). The peptides were 17- or 18-mers, with 11 amino acid (aa) overlaps.

The PepMix SARS-CoV-2 S protein (JPT) covered the residues 1–1273 and consisted of two pools containing 158 (S1) and 157 (S2) peptides, respectively. The S1 and S2 pools (15 mers with 11 aa overlap peptides; > 90% purity) were dissolved in 40 µl of DMSO and then further diluted in PBS.

The SARS-CoV-2 Membrane (M; GenBank MN908947.3, Protein QHD43419.1) and Nucleocapsid (N; GenBank MN908947.3, Protein QHD43423.2) peptide pools consisted of 15 mer peptides with 11 aa overlap (> 70% purity) and spanned the entire protein sequences, were dissolved according the manufacturer’s instructions (Miltenyi Biotech). A list of all individual peptides (except for M and N) and composition of the peptide pools are shown in the Supplementary Tables S3 and S4.

For the stimulation of T cells, cells were incubated with peptide pools: HCoV-NL63 and -OC43 (0.4 µg/ml), SARS-CoV-2 Spike (S1-S2), M and N peptide pools (1 µg/ml). Inactivated HCoV- NL63 and OC43, homologous recombinant S proteins and SARS-CoV-2 S protein, were used at a final concentration of 2.5 µg/ml. The inactivated influenza virus was used at 1.5 µg/ml for each hemagglutinin. The PBMCs were infected with RSV using a multiplicity of infection of 2, for 90 min at 37 °C, 5% CO_2_. After two washes with R10F, the cells were resuspended and used for the ELISpot. Positive and negative controls consisted of CD3 and equimolar amount of DMSO were included in each assay.

### Detection of virus-specific T cells by IFN-γ ELISpot assay

Human IFN-γ ELISpot kits containing 96 well pre-coated plates was purchased from Mabtech and the assay carried out according the manufacturer’s instructions. PBMCs (2 × 10^5^ or 6 × 10^5^ per well) were stimulated for 20 h at 37 °C, 5% CO_2_ in the presence of antigens. Cell incubated with anti-CD3 antibody (Mabtech) or DMSO were used as positive and negative controls, respectively. After development, plates were scanned and spots counted using the ImmunoSpot S6 Ultimate Reader and ImmunoSpot Software (Version 7.0.20.0, Immunospot, CTL).

Data were calculated using the mean of triplicate wells and expressed as spot-forming units or cells (SFU or SFC) per million of PBMCs after subtracting values of DMSO controls. A number > 10 SFU/10^6^ PBMCs, was considered a positive response.

### Detection of NL63 and OC43-specific IgG antibodies

Virus-specific IgGs were measured by enzyme-linked immunosorbent assay (ELISA). Nunc Maxi-Sorp 96 well plate were incubated with 2 µg/ml of inactivated HCoV-NL63 or OC43 in Carbonate/bicabonate buffer (pH 9.6) overnight at 4 °C. Plates were then washed with Phosphate Buffered Saline containing 0.05% Tween 20 (PBS-T) and blocked with 1% BSA for 1 h at room temperature. The serum samples were diluted 1:100 in PBS-T 0.01% BSA and added to the plates for 2 h at room temperature. Virus-specific antibodies were detected using biotinylated anti-human IgG antibody followed by alkaline phosphatase-conjugated streptavidin and p-nitrophenyl phosphate substrate (all from Mabtech). The absorbance of each sample was measured at 405 nm using a microplate reader (Spark, Tecan). The optical density (OD) of the background wells was subtracted from the OD of the antigen wells.

### Flow cytometric analyses

PBMCs (1 × 10^6^ per well) were stimulated for 18 h at 37 °C, 5% CO_2_ with inactivated NL63 and OC43, peptides or recombinant S proteins in the presence of 1 µg/ml each of anti-CD28 and anti-CD49d (BD Biosciences) and Brefeldin A (7 µg/ml; Sigma Aldrich) for the last 5 h of culture. After washing with Phosphate Buffered Saline (PBS), cells where stained for 20 min with LIVE/DEAD Fixable Near-IR stain kit (Molecular Probes) and surface stained with anti-CD14 BV605 (clone M5E2) and anti-CD19 BV605 (clone SJ25C1) antibodies for 30 min at room temperature. Following two washes with PBS, cells were subjected to fixation and permeabilization (Cytofix/Cytoperm; BD Bisociences) and stained with antibodies to CD3 BB515 (clone UCHT-1), CD4 PerCP-Cy5.5 (Clone RPA-4, Biolegend), CD8 APC (Clone RPA-8) and IFN-γ BV421. After 30 min incubation at room temperature, cells were washed twice, resupended in staining buffer and acquired using a BD FortessaX20 (BD Biosciences). For negative and positive controls, cells were incubated with medium and anti-CD3 antibody (BD Biosciences), respectively. Antibodies were purchased from BD Biosciences unless otherwise stated.

At least 200,000 cells per sample were acquired using a BD FortessaX20 (BD Biosciences) and FACS Diva software (version 9.0). Data were analyzed using FlowJo software (Version 10.5.3, BD Bioscience). The data presented correspond to background-subtracted results using the negative controls. The gates were established based on the unstimulated cells and using the FMO approach^55^. Example of the gating strategy is displayed in Supplementary Fig. S2.

### Statistical analyses

Statistical analyses was carried out using GraphPad Prism 8 software (version 8.0.1). The nonparametric Mann–Whitney *U* testing for unpaired samples was used to compare responses between the group of young and older adults. A *p* value of 0.05 or less was considered to be statistically significant.

## Supplementary information

Supplementary Information.

## Data Availability

The datasets generated during and/or analysed during the current study are available from the corresponding author on reasonable request.
