# Supplementary material for: Older adults lack SARS CoV-2 cross-reactive T lymphocytes directed to human coronaviruses OC43 and NL63
Source: Sci Rep. 2020 Dec 8;10:21447. doi: 10.1038/s41598-020-78506-9 (PMC7722724; doi:10.1038/s41598-020-78506-9)
Supplement: Supplementary file 1 — Supplementary Information. [file 41598_2020_78506_MOESM1_ESM.pdf]

Older adults lack SARS CoV-2 cross-reactive T lymphocytes directed to human coronaviruses OC43 and NL63

Giulietta Saletti[1], Thomas Gerlach[1], Janina M. Jansen[1], Antonia Molle[1], Husni Elbahesh[1], Martin Ludlow[1], Wentao Li[2], Berend-Jan Bosch[2], Albert -D.M.E. Osterhaus[1] and Guus F. Rimmelzwaan[1]

[1]Research Center for Emerging Infections and Zoonoses, University of Veterinary Medicine, Hannover, Germany

[2]Virology Section, Infectious Diseases and Immunology Division, Department of Biomolecular Health Sciences, Faculty of Veterinary Medicine, Utrecht University, Utrecht, the Netherlands.

Supplementary Table S1: Demographic characteristics of the study cohort

Young adults

Donor Nr.	Age (years)	sex	HLA Haplotype
1952587	20	F	A*02,*03; B*65, B*51
1952664	19	M	A*01, *24; B* 08 B*61
1952787	22	M	A*03; B*07
1952804	22	M	A*02, A*03; B*07, B*08
1952818	27	M	A*01,A*03; B*08,B*35
1952836	21	M	A*02, A*03; B*13,B*65
1952849	22	M	A*02,A*24; B*07,B*60
1952890	24	F	A*02, A*24; B* 35,B*60
1953028	24	F	A*01, A*02; B* 08, B*44
1953035	21	M	A*02,A*03; B*44, B*51
1953034	23	F	A*30,A*31; B*13, B*35
1953105	20	M	A*01, A*25; B* 08, B*18
1953084	23	M	A*01, A*24; B*07, B*61
1953138	23	F	A*03, A*24; B*07,B*08
1953152	22	F	A*02, A*29; B*37, B*44
1953273	21	M	A*024, A*68; B*07,B*35
1953288	21	F	A*01, A*02; B*08, B*44
1953922	20	M	A*01, A*31; B*62, B*35
1954225	22	F	A*024; B*38,B*44
1954843	23	M	A*02, A*31; B*07,B*62
1954909	21	F	A*02, A*03; B*07, B*51
1954945	23	F	A*03, A*24; B*65, B*18
1956339	23	F	A*2, A*33; B*65, B*35

Older adults

Donor Nr.	Age (years)	sex	HLA Haplotype
1952570	65	M	A*01,A*31; B*8,B*40
1952560	61	M	A*02; B*15, B*18
1952564	64	M	A*01, A*02; B*08, B*44
1952597	69	F	A*02; B *44, B*51
1952637	66	F	A*02, A*31; B*27, B*60
1952718	61	M	A*02, A*68; B*14, B*40
1952706	61	F	A*01, A*02; B*15,B*18
1952731	62	M	A*01, A*68; B*40, B*51
1953101	63	M	A*03; B*18, B*35
1953093	63	M	A*02, A*32; B*07, B*44
1953116	64	M	A*01, A*29; B*27, B*44
1953115	69	M	A*02, A*25; B*18,B*44
1953122	62	M	A*03, A*28; B*07, B*49
1953262	68	M	A*02, A*32; B*35, B*40
1953270	64	M	A*02; B*13, B*51
1953416	65	M	A*02, A*24; B*44, B*13
1953403	66	M	A*02, A*03; B*07, B*27
1954928	70	M	A*02, A*11; B*8, B*37
1955149	65	M	A*24, A*68; B*61, B*44
1956269	62	M	A*02, A*26; B*37, B*38
1956347	68	M	A*24, A*31; B*35, B*60

Supplementary Table S2: IFN-γ ELISpot

Donor Nr.	RSV	NL63	OC43	CD3
1952587	38	351	156	1163
1952664	68	213	112	493
1952787	91	420	255	1213
1952804	40	540	209	1838
1952818	25	9	10	1718
1952836	187	614	474	3162
1952849	52	26	0	2714
1952890	72	233	15	2596
1953028	76	260	14	1696
1953035	36	43	19	399
1953034	352	242	19	3387
1953105	19	50	10	5592
1953084	79	64	1	5021
1953138	447	48	39	8288
1953152	199	261	33	2580
1953273	146	672	510	8000
1953288	244	312	288	7895
1953922	262	111	8	5550
1954225	196	116	51	2455
1954843	231	93	32	3760
1954909	33	3	11	10899
1954945	161	1	0	29147
1956339	66	23	24	2466

Donor Nr.	RSV	NL63	OC43	CD3
1952570	77	59	7	4242
1952560	66	2	0	11928
1952564	12	5	0	7493
1952597	28	96	63	3921
1952637	66	226	46	1656
1952718	18	4	1	5530
1952706	39	171	1	2225
1952731	156	169	12	5596
1953101	46	22	6	6330
1953093	11	11	1	5640
1953116	80	23	0	2696
1953115	9	2	1	3997
1953122	61	34	14	13785
1953262	154	83	2	420
1953270	141	53	41	1447
1953416	5	5	4	1233
1953403	632	25	5	1292
1954928	20	5	0	1764
1955149	353	221	70	12244
1956269	41	24	40	11494
1956347	92	7	11	2591

Supplementary Table S3: Peptides libraries from HCoV-NL63 HCoV-OC43 (Spike protein)

HCoV-NL63-Pool 1 (S1)							
1	MKLFLILLVLPLASCFF	41	GSTLVDGVSRLYQPLRL	81	IYYQHTDINFTATASFG	121	VSNGGNNCTTAVMTYSN
2	LLVLPLASCFFTCNSNA	42	GVSRLYQPLRLTCLWPV	82	DINFTATASFGGSCYVC	122	NCTTAVMTYSNFGICAD
3	ASCFFTCNSNANLSMLQ	43	YQPLRLTCLWPVPGLKS	83	TASFGGSCYVCKPHQVN	123	MTYSNFGICADGSLIPV
4	CNSNANLSMLQLGVPDN	44	TCLWPVPGLKSSTGFVY	84	SCYVCKPHQVNISLNGN	124	GICADGSLIPVRPRNSS
5	LSMLQLGVPDNSSTIVT	45	PGLKSSTGFVYFNATGS	85	PHQVNISLNGNTSVCVR	125	SLIPVRPRNSSDNGISA
6	GVPDNSSTIVTGLLPTH	46	TGFVYFNATGSDVNCNG	86	SLNGNTSVCVRTSHFSI	126	PRNSSDNGISAIITANL
7	STIVTGLLPTHWFCANQ	47	NATGSDVNCNGYQHNSV	87	SVCVRTSHFSIRYIYNR		
8	LLPTHWFCANQSTSVYS	48	VNCNGYQHNSVVDVMRY	88	SHFSIRYIYNRVKSGSP		
9	FCANQSTSVYSANGFFY	49	YQHNSVVDVMRYNLNFS	89	YIYNRVKSGSPGDSSWH		
10	TSVYSANGFFYIDVGNH	50	VDVMRYNLNFSANSLDN	90	KSGSPGDSSWHIYLKSG		
11	NGFFYIDVGNHRSAFAL	51	NLNFSANSLDNLKSGVI	91	DSSWHIYLKSGTCPFSF		
12	DVGNHRSAFALHTGYYD	52	NSLDNLKSGVIVFKTLQ	92	YLKSGTCPFSFSKLNNF		
13	SAFALHTGYYDANQYYI	53	KSGVIVFKTLQYDVLFY	93	CPFSFSKLNNFQKFKTI		
14	TGYYDANQYYIYVTNEI	54	FKTLQYDVLFYCSNSSS	94	KLNNFQKFKTICFSTVE		
15	NQYYIYVTNEIGLNASV	55	DVLFYCSNSSSGVLDTT	95	KFKTICFSTVEVPGSCN		
16	VTNEIGLNASVTLKICK	56	SNSSSGVLDTTIPFGPS	96	FSTVEVPGSCNFPLEAT		
17	LNASVTLKICKFSRNTT	57	VLDTTIPFGPSSQPYYC	97	PGSCNFPLEATWHYTSY		
18	LKICKFSRNTTFDFLSN	58	PFGPSSQPYYCFINSTI	98	PLEATWHYTSYTIVGAL		
19	SRNTTFDFLSNASSSFD	59	SQPYYCFINSTINTTHV	99	HYTSYTIVGALYVTWSE		
20	DFLSNASSSFDCIVNLL	60	FINSTINTTHVSTFVGI	100	IVGALYVTWSEGNSITG		
21	SSSFDCIVNLLFTEQLG	61	NTTHVSTFVGILPPTVR	101	VTWSEGNSITGVPYPVS		
22	IVNLLFTEQLGAPLGIT	62	TFVGILPPTVREIVVAR	102	NSITGVPYPVSGIREFS		
23	TEQLGAPLGITISGETV	63	PPTVREIVVARTGQFYI	103	PYPVSGIREFSNLVLNN		
24	PLGITISGETVRLHLYN	64	IVVARTGQFYINGFKYF	104	IREFSNLVLNNCTKYNI		
25	SGETVRLHLYNVTRTFY	65	GQFYINGFKYFDLGFIE	105	LVLNNCTKYNIYDYVGT		
26	LHLYNVTRTFYVPAAYK	66	GFKYFDLGFIEAVNFNV	106	TKYNIYDYVGTGIIRSS		
27	TRTFYVPAAYKLTKLSV	67	LGFIEAVNFNVTTASAT	107	DYVGTGIIRSSNQSLAG		
28	PAAYKLTKLSVKCYFNY	68	VNFNVTTASATDFWTVA	108	IIRSSNQSLAGGITYVS		
29	TKLSVKCYFNYSCVFSV	69	TASATDFWTVAFATFVD	109	NQSLAGGITYVSNSGNL		
30	CYFNYSCVFSVVNATVT	70	FWTVAFATFVDVLVNVS	110	GITYVSNSGNLLGFKNV		
31	CVFSVVNATVTVNVTTH	71	ATFVDVLVNVSATNIQN	111	NSGNLLGFKNVSTGNIF		
32	NATVTVNVTTHNGRVVN	72	LVNVSATNIQNLLYCDS	112	GFKNVSTGNIFIVTPCN		
33	NVTTHNGRVVNYTVCDD	73	TNIQNLLYCDSPFEKLQ	113	TGNIFIVTPCNQPDQVA		
34	GRVVNYTVCDDCNGYTD	74	LYCDSPFEKLQCEHLQF	114	VTPCNQPDQVAVYQQSI		
35	TVCDDCNGYTDNIFSVQ	75	FEKLQCEHLQFGLQDGF	115	PDQVAVYQQSIIGAMTA		
36	NGYTDNIFSVQQDGRIP	76	EHLQFGLQDGFYSANFL	116	YQQSIIGAMTAVNESRY		
37	IFSVQQDGRIPNGFPFN	77	LQDGFYSANFLDDNVLP	117	GAMTAVNESRYGLQNLL		
38	DGRIPNGFPFNNWFLLT	78	SANFLDDNVLPETYVAL	118	NESRYGLQNLLQLPNFY		
39	GFPFNNWFLLTNGSTLV	79	DNVLPETYVALPIYYQH	119	LQNLLQLPNFYYVSNGG		
40	WFLLTNGSTLVDGVSRL	80	TYVALPIYYQHTDINFT	120	LPNFYYVSNGGNNCTTA		

HCoV-NL63-Pool 2 (S2)					
123	MTYSNFGICADGSLIPV	163	LQENQKILAASFNKAIN	203	VELHTVIPDYVDVNKTL
124	GICADGSLIPVRPRNSS	164	ILAASFNKAINNIVASF	204	IPDYVDVNKTLQEFAQN
125	SLIPVRPRNSSDNGISA	165	NKAINNIVASFSSVNDA	205	VNKTLQEFAQNLPKYVK
126	PRNSSDNGISAIITANL	166	IVASFSSVNDAITQTAE	206	EFAQNLPKYVKPNFDLT
127	NGISAIITANLSIPSNW	167	SVNDAITQTAEAIHTVT	207	PKYVKPNFDLTPFNLTY
128	ITANLSIPSNWTTSVQV	168	TQTAEAIHTVTIALNKI	208	NFDLTPFNLTYLNLSSE
129	IPSNWTTSVQVEYLQIT	169	IHTVTIALNKIQDVVNQ	209	FNLTYLNLSSELKQLEA
130	TSVQVEYLQITSTPIVV	170	ALNKIQDVVNQQGSALN	210	NLSSELKQLEAKTASLF
131	YLQITSTPIVVDCATYV	171	DVVNQQGSALNHLTSQL	211	KQLEAKTASLFQTTVEL
132	TPIVVDCATYVCNGNPR	172	GSALNHLTSQLRHNFQA	212	TASLFQTTVELQGLIDQ
133	CATYVCNGNPRCKNLLK	173	LTSQLRHNFQAISNSIQ	213	TTVELQGLIDQINSTYV
134	NGNPRCKNLLKQYTSAC	174	HNFQAISNSIQAIYDRL	214	GLIDQINSTYVDLKLLN
135	KNLLKQYTSACKTIEDA	175	SNSIQAIYDRLDSIQAD	215	NSTYVDLKLLNRFENYI
136	YTSACKTIEDALRLSAH	176	IYDRLDSIQADQQVDRL	216	LKLLNRFENYIKWPWWV
137	TIEDALRLSAHLETNDV	177	SIQADQQVDRLITGRLA	217	FENYIKWPWWVWLIISV
138	RLSAHLETNDVSSMLTF	178	DQQVDRLITGRLAALNA	218	WPWWVWLIISVVFVVLL
139	ETNDVSSMLTFDSNAFS	179	LITGRLAALNAFVSQVL	219	LIISVVFVVLLSLLVFC
140	SMLTFDSNAFSLANVTS	180	AALNAFVSQVLNKYTEV	220	FVVLLSLLVFCCLSTGC
141	SNAFSLANVTSFGDYNL	181	VSQVLNKYTEVRGSRRL	221	LLVFCCLSTGCCGCCNC
142	ANVTSFGDYNLSSVLPQ	182	KYTEVRGSRRLAQQKIN	222	LSTGCCGCCNCLTSSMR
143	GDYNLSSVLPQRNIRSS	183	GSRRLAQQKINECVKSQ	223	GCCNCLTSSMRGCCDCG
144	SVLPQRNIRSSRIAGRS	184	AQQKINECVKSQSNRYG	224	TSSMRGCCDCGSTKLPY
145	NIRSSRIAGRSALEDLL	185	ECVKSQSNRYGFCGNGT	225	CCDCGSTKLPYYEFEKV
146	IAGRSALEDLLFSKVVT	186	SNRYGFCGNGTHIFSIV	226	TKLPYYEFEKVHVQ
147	LEDLLFSKVVTSGLGTV	187	CGNGTHIFSIVNSAPDG		
148	SKVVTSGLGTVDVDYKS	188	IFSIVNSAPDGLLFLHT		
149	GLGTVDVDYKSCTKGLS	189	SAPDGLLFLHTVLLPTD		
150	VDYKSCTKGLSIADLAC	190	LFLHTVLLPTDYKNVKA		
151	TKGLSIADLACAQYYNG	191	LLPTDYKNVKAWSGICV		
152	ADLACAQYYNGIMVLPG	192	KNVKAWSGICVDGIYGY		
153	AQYYNGIMVLPGVADAE	193	SGICVDGIYGYVLRQPN		
154	IMVLPGVADAERMAMYT	194	GIYGYVLRQPNLVLYSD		
155	VADAERMAMYTGSLIGG	195	LRQPNLVLYSDNGVFRV		
156	MAMYTGSLIGGMVLGGL	196	VLYSDNGVFRVTSRVMF		
157	SLIGGMVLGGLTSAAAI	197	GVFRVTSRVMFQPRLPV		
158	VLGGLTSAAAIPFSLAL	198	SRVMFQPRLPVLSDFVQ		
159	SAAAIPFSLALQARLNY	199	PRLPVLSDFVQIYNCNV		
160	FSLALQARLNYVALQTD	200	SDFVQIYNCNVTFVNIS		
161	ARLNYVALQTDVLQENQ	201	YNCNVTFVNISRVELHT		
162	ALQTDVLQENQKILAAS	202	FVNISRVELHTVIPDYV		

HCoV-OC43 Pool 1 (S1)							
1	MFLILLISLPTAFAVIG	41	FNVYLGMALSHYYVMPL	81	TNHDVVYAQHCFKAPKN	121	LQPINYFDSYLGCVVNA
2	ISLPTAFAVIGDLKCTS	42	MALSHYYVMPLTCNSKV	82	YAQHCFKAPKNFCPCKL	122	FDSYLGCVVNAYNSTAI
3	FAVIGDLKCTSDTSYIN	43	YVMPLTCNSKVKNGFTL	83	KAPKNFCPCKLNGSCVG	123	CVVNAYNSTAISVQTCD
4	LKCTSDTSYINDKDTGP	44	CNSKVKNGFTLEYWVTP	84	CPCKLNGSCVGSGPGKN	124	NSTAISVQTCDLTVGSG
5	TSYINDKDTGPPPISTD	45	NGFTLEYWVTPLTSRQY	85	GSCVGSGPGKNNGIGTC	125	VQTCDLTVGSGYCVDYS
6	KDTGPPPISTDTVDVTN	46	YWVTPLTSRQYLLAFNQ	86	GPGKNNGIGTCPAGTNY	126	TVGSGYCVDYSKNRRSR
7	PISTDTVDVTNGLGTYY	47	TSRQYLLAFNQDGIIFN	87	GIGTCPAGTNYLTCDNL	127	CVDYSKNRRSRGAITTG
8	VDVTNGLGTYYVLDRVY	48	LAFNQDGIIFNAVDCMS	88	AGTNYLTCDNLCTPDPI	128	NRRSRGAITTGYRFTNF
9	LGTYYVLDRVYLNTTLF	49	GIIFNAVDCMSDFMSEI	89	TCDNLCTPDPITFKATG		
10	LDRVYLNTTLFLNGYYP	50	VDCMSDFMSEIKCKTQS	90	TPDPITFKATGTYKCPQ		
11	NTTLFLNGYYPTSGSTY	51	FMSEIKCKTQSIAPPTG	91	FKATGTYKCPQTKSLVG		
12	NGYYPTSGSTYRNMALK	52	CKTQSIAPPTGVYELNG	92	YKCPQTKSLVGIGEHCS		
13	SGSTYRNMALKGSVLLS	53	APPTGVYELNGYTVQPI	93	KSLVGIGEHCSGLAVKS		
14	NMALKGSVLLSRLWFKP	54	YELNGYTVQPIADVYRR	94	GEHCSGLAVKSDYCGGN		
15	SVLLSRLWFKPPFLSDF	55	TVQPIADVYRRKLNLPN	95	LAVKSDYCGGNSCTCRP		
16	LWFKPPFLSDFINGIFA	56	DVYRRKLNLPNCNIEAW	96	YCGGNSCTCRPQAFLGW		
17	FLSDFINGIFAKVKNTK	57	LNLPNCNIEAWLNDKSV	97	CTCRPQAFLGWSADSCL		
18	NGIFAKVKNTKVIKDRV	58	NIEAWLNDKSVPSPLNW	98	AFLGWSADSCLQGDKCN		
19	VKNTKVIKDRVMYSEFP	59	NDKSVPSPLNWERKTFS	99	ADSCLQGDKCNIFANFI		
20	IKDRVMYSEFPAITIGS	60	SPLNWERKTFSNCNFNM	100	GDKCNIFANFILHDVNS		
21	YSEFPAITIGSTFVNTS	61	RKTFSNCNFNMSSLMSF	101	FANFILHDVNSGLTCST		
22	ITIGSTFVNTSYSVVVQ	62	CNFNMSSLMSFIQADSF	102	HDVNSGLTCSTDLQKAN		
23	FVNTSYSVVVQPRTINS	63	SLMSFIQADSFTCNNID	103	LTCSTDLQKANTDIILG		
24	SVVVQPRTINSTQDGYN	64	IQADSFTCNNIDAAKIY	104	LQKANTDIILGVCVNYD		
25	RTINSTQDGYNKLQGLL	65	TCNNIDAAKIYGMCFSS	105	DIILGVCVNYDLYGILG		
26	TQDGYNKLQGLLEVSVC	66	AAKIYGMCFSSITIDKF	106	CVNYDLYGILGQGIFVE		
27	KLQGLLEVSVCQYNMCE	67	MCFSSITIDKFAIPNGR	107	YGILGQGIFVEVNATYY		
28	EVSVCQYNMCEYPQTIC	68	TIDKFAIPNGRKVDLQL	108	GIFVEVNATYYNSWQNL		
29	YNMCEYPQTICHPNLGN	69	IPNGRKVDLQLGNLGYL	109	NATYYNSWQNLLYDSNG		
30	PQTICHPNLGNHRKELW	70	VDLQLGNLGYLQSFNYR	110	SWQNLLYDSNGNLYGFR		
31	PNLGNHRKELWHLDTGV	71	NLGYLQSFNYRIDTTAT	111	YDSNGNLYGFRDYITNR		
32	RKELWHLDTGVVSCLYK	72	SFNYRIDTTATSCQLYY	112	LYGFRDYITNRTFMIRS		
33	LDTGVVSCLYKRNFTYD	73	DTTATSCQLYYNLPAAN	113	YITNRTFMIRSCYSGRV		
34	SCLYKRNFTYDVNADYL	74	CQLYYNLPAANVSVSRF	114	FMIRSCYSGRVSAAFHA		
35	NFTYDVNADYLYFHFYQ	75	LPAANVSVSRFNPSTWN	115	YSGRVSAAFHANSSEPA		
36	NADYLYFHFYQEGGTFY	76	SVSRFNPSTWNKRFGFI	116	AAFHANSSEPALLFRNI		
37	FHFYQEGGTFYAYFTDT	77	PSTWNKRFGFIEDSVFK	117	SSEPALLFRNIKCNYVF		
38	GGTFYAYFTDTGVVTKF	78	RFGFIEDSVFKPRPAGV	118	LFRNIKCNYVFNNSLTR		
39	YFTDTGVVTKFLFNVYL	79	DSVFKPRPAGVLTNHDV	119	CNYVFNNSLTRQLQPIN		
40	VVTKFLFNVYLGMALSH	80	RPAGVLTNHDVVYAQHC	120	NSLTRQLQPINYFDSYL		

HCoV-OC43 Pool 2 (S2)					
126	TVGSGYCVDYSKNRRSR	166	VPFYLNVQYRINGLGVT	206	KAPYVMLNTSIPNLPDF
127	CVDYSKNRRSRGAITTG	167	VQYRINGLGVTMDVLSQ	207	LNTSIPNLPDFKEELDQ
128	NRRSRGAITTGYRFTNF	168	GLGVTMDVLSQNQKLIA	208	NLPDFKEELDQWFKNQT
129	AITTGYRFTNFEPFTVN	169	DVLSQNQKLIANAFNNA	209	EELDQWFKNQTSVAPDL
130	RFTNFEPFTVNSVNDSL	170	NQKLIANAFNNALYAIQ	210	FKNQTSVAPDLSLDYIN
131	PFTVNSVNDSLEPVGGL	171	NAFNNALYAIQEGFDAT	211	VAPDLSLDYINVTFLDL
132	VNDSLEPVGGLYEIQIP	172	LYAIQEGFDATNSALVK	212	LDYINVTFLDLQVEMNR
133	PVGGLYEIQIPSEFTIG	173	GFDATNSALVKIQAVVN	213	TFLDLQVEMNRLQEAIK
134	EIQIPSEFTIGNMEEFI	174	SALVKIQAVVNANAEAL	214	VEMNRLQEAIKVLNQSY
135	EFTIGNMEEFIQTSSPK	175	IQAVVNANAEALNNLLQ	215	LQEAIKVLNQSYINLKD
136	MEEFIQTSSPKVTIDCA	176	ANAEALNNLLQQLSNRF	216	VLNQSYINLKDIGTYEY
137	TSSPKVTIDCAAFVCGD	177	NNLLQQLSNRFGAISAS	217	INLKDIGTYEYYVKWPW
138	TIDCAAFVCGDYAACKS	178	LSNRFGAISASLQEILS	218	GTYEYYVKWPWYVWLLI
139	FVCGDYAACKSQLVEYG	179	AISASLQEILSRLDALE	219	VKWPWYVWLLICLAGVA
140	AACKSQLVEYGSFCDNI	180	LQEILSRLDALEAEAQI	220	VWLLICLAGVAMLVLLF
141	LVEYGSFCDNINAILTE	181	RLDALEAEAQIDRLING	221	LAGVAMLVLLFFICCCT
142	FCDNINAILTEVNELLD	182	AEAQIDRLINGRLTALN	222	LVLLFFICCCTGCGTSC
143	AILTEVNELLDTTQLQV	183	RLINGRLTALNAYVSQQ	223	ICCCTGCGTSCFKKCGG
144	NELLDTTQLQVANSLMN	184	LTALNAYVSQQLSDSTL	224	CGTSCFKKCGGCCDDYT
145	TQLQVANSLMNGVTLST	185	YVSQQLSDSTLVKFSAA	225	KKCGGCCDDYTGYQELV
146	NSLMNGVTLSTKLKDGV	186	SDSTLVKFSAAQAMEKV	226	CDDYTGYQELVIKTSHDD
147	VTLSTKLKDGVNFNVDD	187	KFSAAQAMEKVNECVKS		
148	LKDGVNFNVDDINFSPV	188	AMEKVNECVKSQSSRIN		
149	FNVDDINFSPVLGCLGS	189	ECVKSQSSRINFCGNGN		
150	NFSPVLGCLGSECSKAS	190	SSRINFCGNGNHIISLV		
151	GCLGSECSKASSRSAIE	191	CGNGNHIISLVQNAPYG		
152	CSKASSRSAIEDLLFDK	192	IISLVQNAPYGLYFIHF		
153	RSAIEDLLFDKVKLSDV	193	NAPYGLYFIHFSYVPTK		
154	LLFDKVKLSDVGFVEAY	194	YFIHFSYVPTKYVTARV		
155	KLSDVGFVEAYNNCTGG	195	YVPTKYVTARVSPGLCI		
156	FVEAYNNCTGGAEIRDL	196	VTARVSPGLCIAGDRGI		
157	NCTGGAEIRDLICVQSY	197	PGLCIAGDRGIAPKSGY		
158	EIRDLICVQSYKGIKVL	198	GDRGIAPKSGYFVNVNN		
159	CVQSYKGIKVLPPLLSE	199	PKSGYFVNVNNTWMYTG		
160	GIKVLPPLLSENQISGY	200	VNVNNTWMYTGSGYYYP		
161	PLLSENQISGYTLAATS	201	WMYTGSGYYYPEPITEN		
162	NQISGYTLAATSASLFP	202	GYYYPEPITENNVVVMS		
163	TLAATSASLFPLWTAAA	203	PITENNVVVMSTCAVNY		
164	ASLFPLWTAAAGVPFYL	204	VVVMSTCAVNYTKAPYV		
165	WTAAAGVPFYLNVQYRI	205	CAVNYTKAPYVMLNTSI		

Supplementary Table S4: Peptides libraries from SARS-CoV-2 Spike protein

SARS-CoV-2 Pool 1 (S1)							
1	MFVFLVLLPLVSSQC	41	SSANNCTFEYVSQPF	81	QPTESIVRFPNITNL	121	NGVEGFNCYFPLQSY
2	LVLLPLVSSQCVNLT	42	NCTFEYVSQPFLMDL	82	SIVRFPNITNLCPFG	122	GFNCYFPLQSYGFQP
3	PLVSSQCVNLTTRTQ	43	EYVSQPFLMDLEGKQ	83	FPNITNLCPFGEVFN	123	YFPLQSYGFQPTNGV
4	SQCVNLTTRTQLPPA	44	QPFLMDLEGKQGNFK	84	TNLCPFGEVFNATRF	124	QSYGFQPTNGVGYQP
5	NLTTRTQLPPAYTNS	45	MDLEGKQGNFKNLRE	85	PFGEVFNATRFASVY	125	FQPTNGVGYQPYRVV
6	RTQLPPAYTNSFTRG	46	GKQGNFKNLREFVFK	86	VFNATRFASVYAWNR	126	NGVGYQPYRVVVLSF
7	PPAYTNSFTRGVYYP	47	NFKNLREFVFKNIDG	87	TRFASVYAWNRKRIS	127	YQPYRVVVLSFELLH
8	TNSFTRGVYYPDKVF	48	LREFVFKNIDGYFKI	88	SVYAWNRKRISNCVA	128	RVVVLSFELLHAPAT
9	TRGVYYPDKVFRSSV	49	VFKNIDGYFKIYSKH	89	WNRKRISNCVADYSV	129	LSFELLHAPATVCGP
10	YYPDKVFRSSVLHST	50	IDGYFKIYSKHTPIN	90	RISNCVADYSVLYNS	130	LLHAPATVCGPKKST
11	KVFRSSVLHSTQDLF	51	FKIYSKHTPINLVRD	91	CVADYSVLYNSASFS	131	PATVCGPKKSTNLVK
12	SSVLHSTQDLFLPFF	52	SKHTPINLVRDLPQG	92	YSVLYNSASFSTFKC	132	CGPKKSTNLVKNKCV
13	HSTQDLFLPFFSNVT	53	PINLVRDLPQGFSAL	93	YNSASFSTFKCYGVS	133	KSTNLVKNKCVNFNF
14	DLFLPFFSNVTWFHA	54	VRDLPQGFSALEPLV	94	SFSTFKCYGVSPTKL	134	LVKNKCVNFNFNGLT
15	PFFSNVTWFHAIHVS	55	PQGFSALEPLVDLPI	95	FKCYGVSPTKLNDLC	135	KCVNFNFNGLTGTGV
16	NVTWFHAIHVSGTNG	56	SALEPLVDLPIGINI	96	GVSPTKLNDLCFTNV	136	FNFNGLTGTGVLTES
17	FHAIHVSGTNGTKRF	57	PLVDLPIGINITRFQ	97	TKLNDLCFTNVYADS	137	GLTGTGVLTESNKKF
18	HVSGTNGTKRFDNPV	58	LPIGINITRFQTLLA	98	DLCFTNVYADSFVIR	138	TGVLTESNKKFLPFQ
19	TNGTKRFDNPVLPFN	59	INITRFQTLLALHRS	99	TNVYADSFVIRGDEV	139	TESNKKFLPFQQFGR
20	KRFDNPVLPFNDGVY	60	RFQTLLALHRSYLTP	100	ADSFVIRGDEVRQIA	140	KKFLPFQQFGRDIAD
21	NPVLPFNDGVYFAST	61	LLALHRSYLTPGDSS	101	VIRGDEVRQIAPGQT	141	PFQQFGRDIADTTDA
22	PFNDGVYFASTEKSN	62	HRSYLTPGDSSSGWT	102	DEVRQIAPGQTGKIA	142	FGRDIADTTDAVRDP
23	GVYFASTEKSNIIRG	63	LTPGDSSSGWTAGAA	103	QIAPGQTGKIADYNY	143	IADTTDAVRDPQTLE
24	ASTEKSNIIRGWIFG	64	DSSSGWTAGAAAYYV	104	GQTGKIADYNYKLPD	144	TDAVRDPQTLEILDI
25	KSNIIRGWIFGTTLD	65	GWTAGAAAYYVGYLQ	105	KIADYNYKLPDDFTG	145	RDPQTLEILDITPCS
26	IRGWIFGTTLDSKTQ	66	GAAAYYVGYLQPRTF	106	YNYKLPDDFTGCVIA	146	TLEILDITPCSFGGV
27	IFGTTLDSKTQSLLI	67	YYVGYLQPRTFLLKY	107	LPDDFTGCVIAWNSN	147	LDITPCSFGGVSVIT
28	TLDSKTQSLLIVNNA	68	YLQPRTFLLKYNENG	108	FTGCVIAWNSNNLDS	148	PCSFGGVSVITPGTN
29	KTQSLLIVNNATNVV	69	RTFLLKYNENGTITD	109	VIAWNSNNLDSKVGG	149	GGVSVITPGTNTSNQ
30	LLIVNNATNVVIKVC	70	LKYNENGTITDAVDC	110	NSNNLDSKVGGNYNY	150	VITPGTNTSNQVAVL
31	NNATNVVIKVCEFQF	71	ENGTITDAVDCALDP	111	LDSKVGGNYNYLYRL	151	GTNTSNQVAVLYQDV
32	NVVIKVCEFQFCNDP	72	ITDAVDCALDPLSET	112	VGGNYNYLYRLFRKS	152	SNQVAVLYQDVNCTE
33	KVCEFQFCNDPFLGV	73	VDCALDPLSETKCTL	113	YNYLYRLFRKSNLKP	153	AVLYQDVNCTEVPVA
34	FQFCNDPFLGVYYHK	74	LDPLSETKCTLKSFT	114	YRLFRKSNLKPFERD	154	QDVNCTEVPVAIHAD
35	NDPFLGVYYHKNNKS	75	SETKCTLKSFTVEKG	115	RKSNLKPFERDISTE	155	CTEVPVAIHADQLTP
36	LGVYYHKNNKSWMES	76	CTLKSFTVEKGIYQT	116	LKPFERDISTEIYQA	156	PVAIHADQLTPTWRV
37	YHKNNKSWMESEFRV	77	SFTVEKGIYQTSNFR	117	ERDISTEIYQAGSTP	157	HADQLTPTWRVYSTG
38	NKSWMESEFRVYSSA	78	EKGIYQTSNFRVQPT	118	STEIYQAGSTPCNGV	158	LTPTWRVYSTGSNVF
39	MESEFRVYSSANNCT	79	YQTSNFRVQPTESIV	119	YQAGSTPCNGVEGFN		
40	FRVYSSANNCTFEYV	80	NFRVQPTESIVRFPN	120	STPCNGVEGFNCYFP		

SARS-CoV-2 Pool 2 (S2)							
159	WRVYSTGSNVFQTRA	199	PIKDFGGFNFSQILP	239	NQNAQALNTLVKQLS	279	QIITTDNTFVSGNCD
160	STGSNVFQTRAGCLI	200	FGGFNFSQILPDPSK	240	QALNTLVKQLSSNFG	280	TDNTFVSGNCDVVIG
161	NVFQTRAGCLIGAEH	201	NFSQILPDPSKPSKR	241	TLVKQLSSNFGAISS	281	FVSGNCDVVIGIVNN
162	TRAGCLIGAEHVNNS	202	ILPDPSKPSKRSFIE	242	QLSSNFGAISSVLND	282	NCDVVIGIVNNTVYD
163	CLIGAEHVNNSYECD	203	PSKPSKRSFIEDLLF	243	NFGAISSVLNDILSR	283	VIGIVNNTVYDPLQP
164	AEHVNNSYECDIPIG	204	SKRSFIEDLLFNKVT	244	ISSVLNDILSRLDKV	284	VNNTVYDPLQPELDS
165	NNSYECDIPIGAGIC	205	FIEDLLFNKVTLADA	245	LNDILSRLDKVEAEV	285	VYDPLQPELDSFKEE
166	ECDIPIGAGICASYQ	206	LLFNKVTLADAGFIK	246	LSRLDKVEAEVQIDR	286	LQPELDSFKEELDKY
167	PIGAGICASYQTQTN	207	KVTLADAGFIKQYGD	247	DKVEAEVQIDRLITG	287	LDSFKEELDKYFKNH
168	GICASYQTQTNSPRR	208	ADAGFIKQYGDCLGD	248	AEVQIDRLITGRLQS	288	KEELDKYFKNHTSPD
169	SYQTQTNSPRRARSV	209	FIKQYGDCLGDIAAR	249	IDRLITGRLQSLQTY	289	DKYFKNHTSPDVDLG
170	QTNSPRRARSVASQS	210	YGDCLGDIAARDLIC	250	ITGRLQSLQTYVTQQ	290	KNHTSPDVDLGDISG
171	PRRARSVASQSIIAY	211	LGDIAARDLICAQKF	251	LQSLQTYVTQQLIRA	291	SPDVDLGDISGINAS
172	RSVASQSIIAYTMSL	212	AARDLICAQKFNGLT	252	QTYVTQQLIRAAEIR	292	DLGDISGINASVVNI
173	SQSIIAYTMSLGAEN	213	LICAQKFNGLTVLPP	253	TQQLIRAAEIRASAN	293	ISGINASVVNIQKEI
174	IAYTMSLGAENSVAY	214	QKFNGLTVLPPLLTD	254	IRAAEIRASANLAAT	294	NASVVNIQKEIDRLN
175	MSLGAENSVAYSNNS	215	GLTVLPPLLTDEMIA	255	EIRASANLAATKMSE	295	VNIQKEIDRLNEVAK
176	AENSVAYSNNSIAIP	216	LPPLLTDEMIAQYTS	256	SANLAATKMSECVLG	296	KEIDRLNEVAKNLNE
177	VAYSNNSIAIPTNFT	217	LTDEMIAQYTSALLA	257	AATKMSECVLGQSKR	297	RLNEVAKNLNESLID
178	NNSIAIPTNFTISVT	218	MIAQYTSALLAGTIT	258	MSECVLGQSKRVDFC	298	VAKNLNESLIDLQEL
179	AIPTNFTISVTTEIL	219	YTSALLAGTITSGWT	259	VLGQSKRVDFCGKGY	299	LNESLIDLQELGKYE
180	NFTISVTTEILPVSM	220	LLAGTITSGWTFGAG	260	SKRVDFCGKGYHLMS	300	LIDLQELGKYEQYIK
181	SVTTEILPVSMTKTS	221	TITSGWTFGAGAALQ	261	DFCGKGYHLMSFPQS	301	QELGKYEQYIKWPWY
182	EILPVSMTKTSVDCT	222	GWTFGAGAALQIPFA	262	KGYHLMSFPQSAPHG	302	KYEQYIKWPWYIWLG
183	VSMTKTSVDCTMYIC	223	GAGAALQIPFAMQMA	263	LMSFPQSAPHGVVFL	303	YIKWPWYIWLGFIAG
184	KTSVDCTMYICGDST	224	ALQIPFAMQMAYRFN	264	PQSAPHGVVFLHVTY	304	PWYIWLGFIAGLIAI
185	DCTMYICGDSTECSN	225	PFAMQMAYRFNGIGV	265	PHGVVFLHVTYVPAQ	305	WLGFIAGLIAIVMVT
186	YICGDSTECSNLLLQ	226	QMAYRFNGIGVTQNV	266	VFLHVTYVPAQEKNF	306	IAGLIAIVMVTIMLC
187	DSTECSNLLLQYGSF	227	RFNGIGVTQNVLYEN	267	VTYVPAQEKNFTTAP	307	IAIVMVTIMLCCMTS
188	CSNLLLQYGSFCTQL	228	IGVTQNVLYENQKLI	268	PAQEKNFTTAPAICH	308	MVTIMLCCMTSCCSC
189	LLQYGSFCTQLNRAL	229	QNVLYENQKLIANQF	269	KNFTTAPAICHDGKA	309	MLCCMTSCCSCLKGC
190	GSFCTQLNRALTGIA	230	YENQKLIANQFNSAI	270	TAPAICHDGKAHFPR	310	MTSCCSCLKGCCSCG
191	TQLNRALTGIAVEQD	231	KLIANQFNSAIGKIQ	271	ICHDGKAHFPREGVF	311	CSCLKGCCSCGSCCK
192	RALTGIAVEQDKNTQ	232	NQFNSAIGKIQDSLS	272	GKAHFPREGVFVSNG	312	KGCCSCGSCCKFDED
193	GIAVEQDKNTQEVFA	233	SAIGKIQDSLSSTAS	273	FPREGVFVSNGTHWF	313	SCGSCCKFDEDDSEP
194	EQDKNTQEVFAQVKQ	234	KIQDSLSSTASALGK	274	GVFVSNGTHWFVTQR	314	CCKFDEDDSEPVLKG
195	NTQEVFAQVKQIYKT	235	SLSSTASALGKLQDV	275	SNGTHWFVTQRNFYE	315	DEDDSEPVLKGVKLHYT
196	VFAQVKQIYKTPPIK	236	TASALGKLQDVVNQN	276	HWFVTQRNFYEPQII		
197	VKQIYKTPPIKDFGG	237	LGKLQDVVNQNAQAL	277	TQRNFYEPQIITTDN		
198	YKTPPIKDFGGFNFS	238	QDVVNQNAQALNTLV	278	FYEPQIITTDNTFVS		

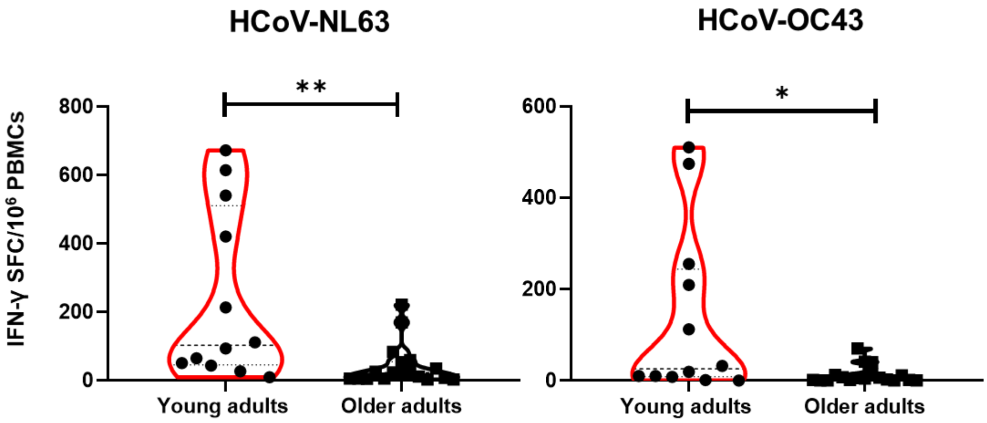

Supplementary Figure S1. HCoV-NL63 and HCoV-OC43-specific immunity in young and older male adults. HCoV-specific T cells response measured as frequency of IFN-γ spot forming cells (SFU) after stimulation of PBMCs isolated from young (n=12; red outline) and older adults (n=18; black outline) with inactivated HCoV-NL63 and HCoV-OC43. Data were background-subtracted and expressed as SFU/10^6 PBMCs. Dotted lines represented the median. Each dot and symbol represent a single subject. Statistical comparison between the two groups was performed using non-parametric Mann-Whitney test for unpaired samples (**p<0.005; *p<0.05).

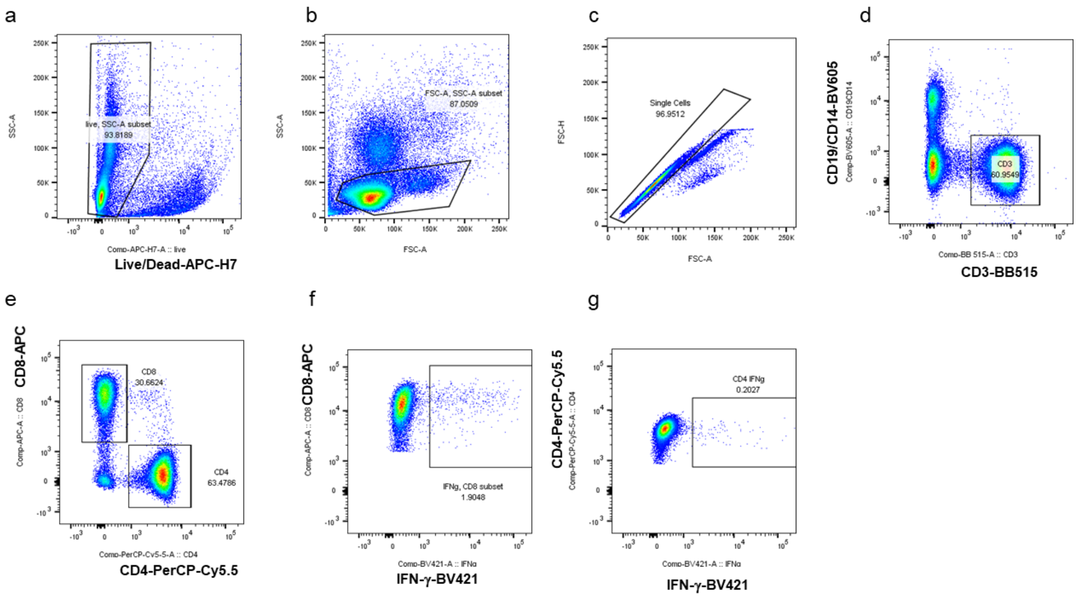

Supplementary Figure S2. Gating strategy for detection of HCoVs-specific IFN-γ+ T cells upon stimulation of PBMCs. (a) Live cells were identified followed by (b) lymphocytes selection and (c) doublets discrimination. (d) The CD3+ T cells were divided into (e) CD4+ and CD8+ T cells subsets and the percentage of IFN-γ+ cells were calculated for each subset. Data were analyzed and images created using FlowJo software (Version 10.5.3, BD Bioscience)
